# Microbiological Quality Assessment of Popular Fresh Date Samples Available in Local Outlets of Dhaka City, Bangladesh

**DOI:** 10.1155/2018/7840296

**Published:** 2018-08-30

**Authors:** Rausan Zamir, A. B. M. Nazmul Islam, Anisur Rahman, Sunzid Ahmed, M. Omar Faruque

**Affiliations:** ^1^Department of General Educational Development, Daffodil International University, Dhaka, Bangladesh; ^2^Uppsala University, Sweden; ^3^Centre for Advanced Research in Science, University of Dhaka, Bangladesh; ^4^Department of Nutrition and Food Technology, Jessore University of Science and Technology, Jessore, Bangladesh

## Abstract

It is evident that date fruits provide a wide variety of essential nutrients which impart potential human health benefits. In Bangladesh, the popularity of date and its consumption surge few-fold during Ramadan among Muslims owing to the profound emotion related to religious belief that breaking of dawn-to-dusk fasting with dates is fulfilling a Sunnah. The present study aimed to explore the microbiological quality of the five most popular fresh date samples (Nagal, Dhapas, Boroi, Morium, and Tunisia) purchased from different locations of Dhaka City, Bangladesh. Among 25 samples tested, 23 were observed to carry 3.30-5.65 Log CFU/gm aerobic bacteria and 3.30-5.36 Log CFU/gm yeasts and molds population. Coliform bacteria and* Escherichia coli* were not found in any of the samples analyzed in this study. However, except Nagal and Boroi from Mohammadpur and Mirpur, respectively, none of the samples were found safe to consume according to the microbiological grade recommended by Woolworths Quality Assurance Standard (WQAS), 2009, as fresh dates with microbial load can cause food poisoning or even foodborne intoxication. Date samples with less or no processing is responsible for this contamination which can be subsided and eliminated by appropriate handling and hygiene practices during postharvest processing.

## 1. Introduction

The dates (*Phoenix dactylifera* L.) are sugar rich fresh fruits mainly cultivated in Middle East countries and used as human food in almost all over the world. There are more than 2000 different varieties of dates available for about 8 months of a year which are consumed by people with high religious value [1]. The chemical composition and quality of dates are dependent on several factors such as the types of cultivar, climatic and farming conditions, and pre- and postharvest practices. Varying with the size and types of cultivars each date retains 20-70 calories [2] and the chemical components of dates are always dominated by fructose and glucose sugar, also retaining moisture, fibers, proteins, fats, minerals, and vitamins (Table 1).

The quality and shelf life of dates are also determined by the types and levels of microorganisms associated. High annual loss of date quality is reported due to microbial invasion, and the rate of contamination is dependent on several critical factors such as cultivation weather, size, ripening stage, postharvest conditions like hygienic practices during processing and handling, storage, and transportation [3]. Generally fresh dates may carry nonpathogenic epiphytic microorganisms, but presence of pathogens/microorganisms, including* Escherichia coli*,* Salmonella* spp.,* Streptococcus* spp.,* Staphylococcus aureus*,* Bacillus cereus*,* Aspergillus* spp.,* Alternaria* spp., and* Fusarium* spp. [2, 4, 5], in date flesh in a level higher than the satisfactory limit set by the responsible regulatory bodies is a potential public health risk from the food safety view point. In addition to bacteria and molds, sometimes the association of osmotolerant yeasts species in date flesh may occur even after packaging and refrigeration, being marked as another important cause that can affect the quality of dates [2]. Bacterial growth is facilitated due to the high moisture content of date flesh; on the other hand growth of molds becomes prominent when the dates are dried and stored [6]. Spoilage of fresh fruits like dates can be initiated when these microorganisms grow in a range from 6.0 Log CFU/gm to higher on the surface or inside the fruits. 

Therefore, possible ways of microbial contamination should be avoided to maintain the quality and safety of dates. This study was conducted to analyze the microbiological quality of ripen dates samples sold in the local markets of Dhaka City.

## 2. Materials and Methods

### 2.1. Sample Collection

**Figure 1:**
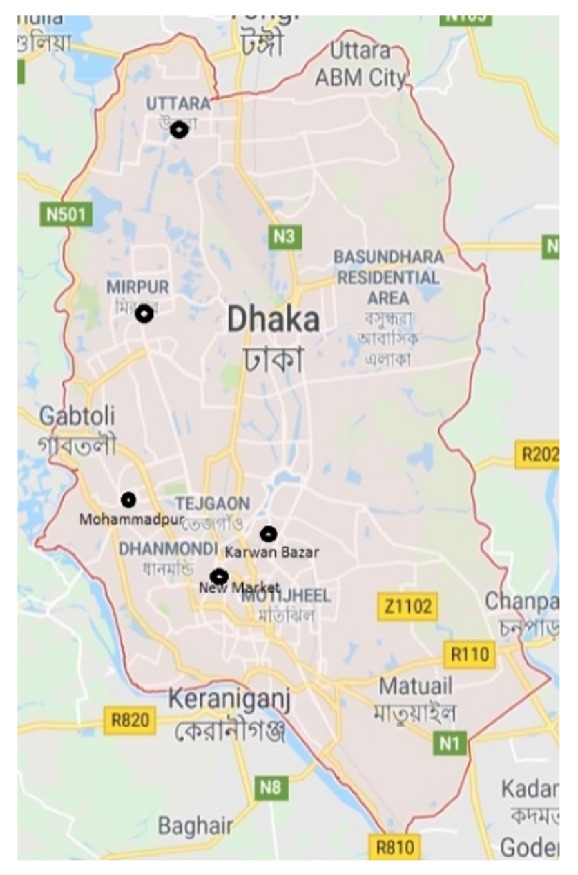
Sampling location of the study area in Dhaka City.

Five varieties of popular fresh date samples (Nagal, Dhapas, Boroi, Morium, and Tunisia) were purchased from different local outlets (Mohammadpur, New Market, Mirpur, Karwan Bazar, and Uttara) of Dhaka City, Bangladesh (Figure 1), during May-July 2017.

During the collection time the ambient environmental temperature was fluctuating between 21 and 36.8°C (Bangladesh Meteorological Department, Climate Division, Agargaon, Dhaka). The samples were collected by maintaining aseptic manners in sterile plastic sample bags (3M, USA) and carried to the laboratory within 4 hours and stored at 4°C until microbiological analysis.

### 2.2. Microbiological Analysis

10 gm deseeded fresh date flesh was weighed and homogenized with 90 ml sterile normal saline (0.85% NaCl; Active Fine Chemicals Ltd., Bangladesh) in sterile stomacher bag. Following serial dilution technique, decimal dilutions were prepared, and 0.1 ml of sample from appropriate dilution was added on different nonselective and selective culture media, followed by microbiological analysis by surface plate method. Nutrient Agar (Oxoid, England), Potato Dextrose Agar (HiMedia, India), and MacConkey Agar (Oxoid, England) were the culture media of choice for aerobic plate count (APC), yeasts and molds count (YMC), total coliform count (TCC), and* E. coli *count.

## 3. Results and Discussion

All of the Date samples of all types (Nagal, Dhapas, Boroi, Morium, and Tunisia) collected from New Market and Karwan Bazar area were found containing significant amount of TABC (aerobic bacteria) ranging from 4.34 to 5.65 and from 4.08 to 4.58 log CFU/ gm, respectively. However, just two samples of Nagal and Morium dates from Mohammadpur outlet were minimum TABC containing dates (<1.0 log CFU/ gm), leaving others contaminated with TABC in range of 3.95-4.51 log CFU/ gm of the same outlet. Nagal dates from Mirpur outlet were also found less contaminated with TABC (<1.0 log CFU/ gm). Date samples of Borai origin were minimally contaminated with TABC in Mirpur and Uttara outlets (<1.0 log CFU/ gm). Dhapas, Morium, and Tunisia of Mirpur outlet and, except Borai, all samples of Uttara outlet were contaminated significantly with TABC (Table 2).

Except New Market outlet, Dhapas dates from all remaining markets under investigation (Mohammadpur, Mirpur, Karwan Bazar, and Uttara) were found least containing yeast and molds (<1.0 log CFU/ gm). New Market area in terms of yeast and mold count may cause a notorious scratch, as in our investigation it was identified as significant yeast and mold containing region (3.60-5.36 log CFU/ gm) for all types of samples. On the contrary, just two types of dates, namely, Tunisia (4.36 log CFU/ gm) and Nagal (4.89 log CFU/ gm), were yeast and mold containing in Karwan Bazar area (Table 2).

Presence of TCC and* E. coli* were menace in all types of dates provided by vendors of all of the study areas in Dhaka City (Mohammadpur, Mirpur, Karwan Bazar, and Uttara) (Table 2).

After microbiological quality analysis it was found that, except the Nagal and Boroi from Mohammadpur and Mirpur outlet, respectively, all the samples were contaminated with unsatisfactory level of aerobic bacteria and/or yeasts and molds according to the guidelines provided by WQAS, 2009. Although date flesh is reported to contain high sugar content and antimicrobial components like tannins (around 2.5%), which is inhibitory to a certain load of both bacteria and fungi [8–11], these levels of microbial contamination (Table 2) are clearly indicating poor hygiene and handling practices during postharvest processing of the fruits. According to the visual observation during sample collection, the unsanitary conditions of the marketing areas are considered to contribute airborne microorganisms including fungal spores to the dates samples displayed openly.

Windy and dry months during cultivation may also carry toxigenic fungal spores, which can find ways to survive and germinate even in low temperature storage condition after postharvest processing [2]. Germination and multiplication of these fungal spores can introduce mycotoxins such as aflatoxins, ochratoxin, and enniatin, which are considered as a serious risk factor challenging the quality and safety of dates [12].

Our research work on microbial contamination in date samples was essentially in imported date products found in local outlets of Dhaka City, and here we found that none of the samples were contaminated with coliform bacteria and* E. coli*. Analysis reveals that throughout the cultivation, harvesting, processing, storage, and transportation process there are less possible routes open for the entrance of* E. coli* and coliform bacteria. Apart from this, the hot summer weather during the sample collection days and antibacterial activity of date flesh itself could suppress the growth or survival of these pathogenic bacteria. To prevent or reduce the microbial invasion into dates sold in open markets in developing countries like Bangladesh, the hygiene education or sanitary knowledge of vendors should be amended.

It was highly unlikely for us to have first-hand data on microbial contamination on dates related to the cultivated plants from where date samples came and therefore we did restrict our investigation to imported date samples. Therefore, subsequent comparison between dates during harvesting and after importing and selling was not outcome in this study.

## 4. Conclusion

In a horrifying account, 92% (23 out of 25) of date samples collected were found to have microbial contamination in ranges higher than acceptable limit recommended by WQAS. Presence of microbial contamination in fresh date is due to less or even no further processing as just tap water washing of ripen dates is done before human consumption. Considering risks associated with food safety, minimization and elimination of microbial load should be ensured by not compromising this problem. Keeping that in mind, the following is recommended: (i) in addition to ensuring proper hygienic practices during postharvest processing, treating the packaged samples with *γ* irradiation before storage and transportation as an effective way to mitigate the possible microbial risks is required [3]; (ii) washing the dates with food grade sanitizers before consumption is advised to assure further safety especially when the dates are purchased from local open outlets; and (iii) as it is a preliminary study to assess microbial load and contamination in food, specially date samples during Ramadan, to have scientific data, research should be extended regarding this issue.

## Figures and Tables

**Table 1:** Chemical composition of dates [2, 7].

**Composition**	**Amount (**%**)**
Total sugars	35-88
Moisture	7.2-31.9
Fibers	6.4-11.5
Proteins	2.3-5.6
Fats	0.2-0.4

**Table 2:** Microbiological analysis of fresh date flesh samples.

**Name of market**	**Name of sample**	**Microbiological parameters (log CFU/gm)**			
		TABC	TCC	*E. coli*	Yeast & Molds
**Mohammadpur**	Nagal	<1.0*∗*	<1.0	<1.0	3.0
	Dhapas	4.51	<1.0	<1.0	<1.0
	Boroi	3.95	<1.0	<1.0	3.30
	Morium	<1.0	<1.0	<1.0	4.69
	Tunisia	4.20	<1.0	<1.0	3.70

**New market**	Nagal	5.65	<1.0	<1.0	5.36
	Dhapas	5.23	<1.0	<1.0	3.60
	Boroi	4.00	<1.0	<1.0	3.78
	Morium	4.34	<1.0	<1.0	3.48
	Tunisia	4.81	<1.0	<1.0	3.78

**Mirpur**	Nagal	<1.0	<1.0	<1.0	4.93
	Dhapas	3.95	<1.0	<1.0	<1.0
	Boroi	<1.0	<1.0	<1.0	<1.0
	Morium	3.70	<1.0	<1.0	3.60
	Tunisia	4.85	<1.0	<1.0	4.88

**Karwan Bazar**	Nagal	4.18	<1.0	<1.0	4.89
	Dhapas	4.45	<1.0	<1.0	<1.0
	Boroi	4.08	<1.0	<1.0	<1.0
	Morium	4.11	<1.0	<1.0	<1.0
	Tunisia	4.58	<1.0	<1.0	4.36

**Uttara**	Nagal	3.30	<1.0	<1.0	3.30
	Dhapas	4.53	<1.0	<1.0	<1.0
	Boroi	<1.0	<1.0	<1.0	3.30
	Morium	3.85	<1.0	<1.0	3.48
	Tunisia	3.48	<1.0	<1.0	3.70

*∗*<1.0: below detection limit; lowest detection limit 1.0 Log CFU/gm

## Data Availability

The data used in the manuscript are available from the corresponding author upon request.
